# *In vitro* test of the novel antibiotic lefamulin alone and in combination with doxycycline against *Mycoplasma genitalium*

**DOI:** 10.1128/aac.01346-24

**Published:** 2024-12-13

**Authors:** Kirsten Salado-Rasmussen, Christina Nørgaard, Thomas Roland Pedersen, Susanne Paukner, Jørgen Skov Jensen

**Affiliations:** 1 Department of Bacteria, Parasites and Fungi, Statens Serum Institut 4326 , Copenhagen, Denmark; 2 Department of Dermato-Venereology, Bispebjerg University Hospital 53146 , Copenhagen, Denmark; 3 Department of Clinical Medicine, University of Copenhagen 4321 , Copenhagen, Denmark; 4 Nabriva Therapeutics GmbH 210810 , Vienna, Austria; Bill & Melinda Gates Medical Research Institute, Cambridge, Massachusetts, USA

**Keywords:** *Mycoplasma genitalium*, antibiotic resistance, pleuromutilin, lefamulin

## Abstract

*Mycoplasma genitalium*, a sexually transmitted bacterium, is a significant cause of urethritis in men and various reproductive tract infections in women, including cervicitis, pelvic inflammatory disease, endometritis, and potentially infertility. Treatment has become increasingly challenging due to the emergence of resistance to both first-line (azithromycin) and second-line (moxifloxacin) antibiotics. The need for new treatment options is critical. This study evaluates the *in vitro* efficacy of the novel antibiotic lefamulin against 54 *M*. *genitalium* isolates, including highly resistant variants. Additionally, the potential synergistic effects of combining lefamulin with doxycycline were assessed in eight selected isolates. Lefamulin exhibited strong antibacterial activity across all tested isolates, with minimal inhibitory concentrations (MICs) ranging from 0.0005 to 0.064 µg/mL. Minimal bactericidal concentrations ranged from 0.001 to 0.128 µg/mL and were equal to the MIC in 40 of 54 isolates and within two- and fourfold MIC in the rest of the isolates. Notably, lefamulin’s MIC values were significantly lower than those of azithromycin, doxycycline, and moxifloxacin, underscoring its potent efficacy. Checkerboard assays revealed no antagonistic interaction between lefamulin and doxycycline, with some additive effects observed in certain isolates. These findings highlight lefamulin’s potential as a highly effective treatment for *M. genitalium* infections, particularly those involving multi-drug-resistant strains. Given the increasing rates of resistance and the limitations of current therapies, lefamulin may represent a promising new option for managing this challenging pathogen. Further clinical studies are warranted to confirm these *in vitro* results and explore the therapeutic potential of lefamulin in combination with doxycycline.

## INTRODUCTION

*Mycoplasma genitalium* is a sexually transmitted bacterium that can cause urethritis in men and cervicitis, pelvic inflammatory disease, endometritis, and possibly infertility in women (1–6). Treatment of *M. genitalium* is increasingly problematic due to resistance development to both first-line (azithromycin) and second-line (moxifloxacin) treatment (4). In a recent meta-analysis, 50% of *M. genitalium* strains carried macrolide-associated mutations, and the prevalence of fluoroquinolone resistance-associated mutations was almost 8% globally (7). Furthermore, dual-class resistance-associated mutations have been reported (7), and third-line pristinamycin (4) has limited availability and is only 75% effective in a clinical setting (8). Macrolide resistance is caused by a single-base mutation in positions A2058 or A2059 (*Escherichia coli* numbering) in region V of the 23S rRNA gene (9), which is only present in one copy in the genome of *M. genitalium*. Thus, macrolide susceptibility can change in one mutational event in *M. genitalium* in contrast to other bacterial pathogens that often harbor multiple copies of the 23S rRNA gene. Due to the increasing rates of macrolide resistance, moxifloxacin has become more widely used even though the drug has been associated with severe side effects and is contraindicated during pregnancy, in adolescents, and patients with a wide range of comorbidities. Given the increasing rates of resistance and the limitations of the current therapies, there is an urgent need to explore new antimicrobials.

Lefamulin, a semisynthetic pleuromutilin antimicrobial that is the first of its class approved for the oral and intravenous treatment of community-acquired bacterial pneumonia (10–12), is also under evaluation for the treatment of sexually transmitted infections as it has demonstrated potent *in vitro* activity against *Neisseria gonorrhoeae, M. genitalium,* and *Chlamydia trachomatis* in earlier studies (13–17), as well as good tissue distribution into urogenital tissues (18).

We tested the *in vitro* antibacterial and bactericidal effect of the novel antibiotic lefamulin on 54 isolates of *M. genitalium*. Additionally, we tested the synergy between lefamulin and doxycycline in eight isolates of *M. genitalium*.

## MATERIALS AND METHODS

### *M. genitalium* isolates

A collection of 54 *M*. *genitalium* isolates from 49 individuals was tested and included the *M. genitalium* G37 type strain and an early passage of the M30 strain isolated by David Taylor-Robinson in 1980 (19). All isolates were tested for macrolide-associated mutations in region V of the 23S rRNA gene and characterized concerning the quinolone-resistance-determining-region of ParC (primarily amino acid positions S83 and D87; *M. genitalium* numbering) and GyrA (Table 1). The isolates comprised 31 macrolide-resistant and 23 susceptible isolates, with 15 moxifloxacin-resistant isolates, 14 of which were also macrolide resistant.

**TABLE 1:** *In vitro* minimal inhibitory concentration and minimal bactericidal concentration of 54 *M*. *genitalium* isolates from 49 individuals^*a*^^,^^*b*^

Strain^*c*^	23s rRNA gene mutation	ParC mutation	GyrA mutation	LEF MIC (µg/mL)	LEF MBC (µg/mL)	AZM MIC (µg/mL)	DOX MIC (µg/mL)	MXF MIC (µg/mL)
G37	WT	WT	WT	0.002	0.002	≤0.001	0.5	0.125
M2282	WT	WT	WT	0.001	0.001	≤0.001	0.5	0.125
M2300	WT	WT	WT	0.001	0.001	≤0.001	0.5	0.125
M2321	WT	WT	WT	0.0005	0.001	≤0.001	1	0.125
M2341	WT	P62S (NS); c234t silent	t378c silent	0.002	0.002	≤0.001	2	0.063
M30	WT	WT	WT	0.001	0.001	≤0.001	0.25	0.125
M6090^1^	WT	WT	a288g silent	0.002	0.002	≤0.001	0.125	0.125
M6151^1^	WT	WT	a288g silent	0.002	0.002	0.008	1	0.125
M6257	A2058G	WT	WT	0.016	0.016	128	1	0.125
M6270	A2059G	c186t silent	WT	0.032	0.032	64	0.5	0.125
M6271	A2058G	S83N (NS)	WT	0.008	0.008	64	0.5	0.25
M6280	WT	P62S (NS); c234t silent	WT	0.001	0.002	≤0.001	0.5	0.03
M6281	WT	WT	WT	0.002	0.002	0.002	1	0.125
M6282	WT	P62S (NS); c234t silent	WT	0.001	0.001	≤0.001	0.5	0.125
M6283	WT	A69T (NS)	WT	0.002	0.002	≤0.001	2	0.125
M6284	WT	WT	WT	0.002	0.002	0.004	1	0.125
M6287	WT	D87Y	WT	0.001	0.002	0.002	0.5	16
M6302	A2058C	WT	WT	0.016	0.016	>8	0.25	0.125
M6303	A2059G	WT	WT	0.016	0.032	64	2	0.25
M6312^1^	WT	WT	a288g silent	0.002	0.002	0.004	0.5	0.125
M6315	WT	WT	WT	0.004	0.004	0.002	2	0.063
M6320	A2059G	WT	WT	0.032	0.064	32	0.5	0.063
M6321	A2058G	WT	WT	0.004	0.004	128	0.5	0.063
M6327	WT	WT	ND	0.001	0.004	0.016	0.5	0.125
M6375	WT	P62S (NS); c234t silent	WT	0.004	0.004	≤0.001	0.5	0.063
M6475	WT	WT	H71Y (NS)	0.002	0.002	≤0.001	1	0.063
M6489	A2059G	S83I	D99N	0.064	0.128	64	2	16
M6592^2^	A2059G	WT	WT	0.016	0.016	16	0.25	0.063
M6594^2^	A2059G	WT	WT	0.008	0.032	32	0.5	0.03
M6595^2^	A2059G	WT	WT	0.016	0.032	64	0.25	0.063
M6604	A2058G	WT	WT	0.016	0.016	128	1	0.125
M6712	A2059G	S83I	WT	0.064	0.128	64	1	8
M6714	A2058G	D87N	D99N	0.008	0.008	128	1	1
M6735	A2059G	S83I	D99N	0.032	0.064	32	1	8
M6926	A2058T	S83I	WT	0.008	0.016	16	1	4
M6927	A2059G	S83I	D99N	0.064	0.128	64	2	4
M6928	A2059G	WT	WT	0.008	0.008	32	0.5	0.125
M6957	A2058G	P62S (NS)	WT	0.004	0.004	64	0.5	0.063
M6958	A2059G	S83I	WT	0.032	0.032	32	2	0.5
M6959	A2058G	S83R	WT	0.016	0.016	64	2	1
M6965	A2058G	D87N	WT	0.002	0.002	64	1	4
M6984	A2058G	D87N	ND	0.008	0.016	64	1	0.5
M7006	A2059G	S83I	M95I	0.064	0.064	64	2	4
M7026^3^	A2059G	S83I	WT	0.032	0.032	32	0.5	1
M7027^3^	A2059G	S83I	WT	0.064	0.064	64	1	1
M7029	A2059G	S83I	WT	0.064	0.064	64	2	4
M7042	A2059G	WT	WT	0.008	0.008	16	0.5	0.125
M7043	WT	WT	WT	0.001	0.001	≤0.001	0.125	0.125
M7044	WT	P62S (NS)	WT	0.002	0.002	<0.001	0.25	0.063
M7045	WT	WT	WT	0.001	0.001	0.002	0.5	0.25
M7046	WT	WT	c336t silent	0.001	0.001	0.002	0.25	0.25
M7048	A2058G	WT	WT	0.004	0.004	64	0.5	0.063
M7049	A2059G	WT	WT	0.016	0.016	32	0.5	0.125
M7050	A2059G	WT	WT	0.004	0.004	64	0.5	0.125

^*a*^ Macrolide resistance-mediating mutations in the 23S rRNA gene according to *Escherichia coli* numbering. ParC mutations leading to moxifloxacin resistance according to *M. genitalium* numbering.

^*b*^ LEF, lefamulin; AZM, azithromycin; DOX, doxycycline; MXF, moxifloxacin; WT, wild type; NS, non-significant mutation; and ND, not done.

^*c*^ Isolates originating from the same individual are marked with superscript numbers 1–3.

### *M. genitalium* minimal inhibitory concentration and minimal bactericidal concentration determination

Minimal inhibitory concentrations (MICs) and minimal bactericidal concentrations (MBCs) were determined for 54 *M*. *genitalium* isolates from 49 patients carrying genetically distinct *M. genitalium* strains. MIC for lefamulin was determined in the range from 0.0005 to 0.125 µg/mL by serial twofold dilutions of antimicrobial added to a Vero cell culture supporting the growth of *M. genitalium* as previously described (20). The Vero cell culture system allows MIC determination also for *M. genitalium* strains that are not adapted to growth in mycoplasma medium. This method is in agreement with the CLSI broth dilution assay; however, macrolide MICs tend to be slightly higher with the Vero cell culture method (21). After a 3-week incubation period, the growth of bacteria was determined using quantitative PCR (qPCR) (22). All quantifications were performed without duplicate testing due to the high reproducibility of the method. Previous studies provided information on the MICs for azithromycin, doxycycline, and moxifloxacin. MBC was determined by transferring 1/10 of the medium used for MIC determination into a Vero cell suspension without antibiotics, and the resulting 10-fold dilution of lefamulin diluted the antibiotic beyond the MIC to allow the re-growth of live *M. genitalium* cells. Growth was determined by qPCR after 4 weeks of incubation.

### Determination of synergy between lefamulin and doxycycline against *M. genitalium*

Checkerboards representing eight-by-eight twofold dilutions of lefamulin and doxycycline, respectively, with the mid-point concentration representing the doxycycline and lefamulin MICs, respectively, were prepared in Vero cell suspensions. *M. genitalium* cells were grown for 3 weeks before growth was determined by qPCR. Results were expressed as the fractional inhibitory concentration index (FICI). Synergy was assumed when FICI was ≤0.5, antagonism when FICI was >4.0, and indifference when FICI was >0.5–4.0 (23, 24).

### Statistical methods

The Mann-Whitney test was applied for comparisons between independent groups, and the Wilcoxon signed-rank test was used for comparisons across different antibiotics. Differences in proportions were compared with Fisher’s exact test. The statistical analyses were performed using R.

## RESULTS

### *In vitro* activity (MIC and MBC) of lefamulin and comparators

**Fig 1:**
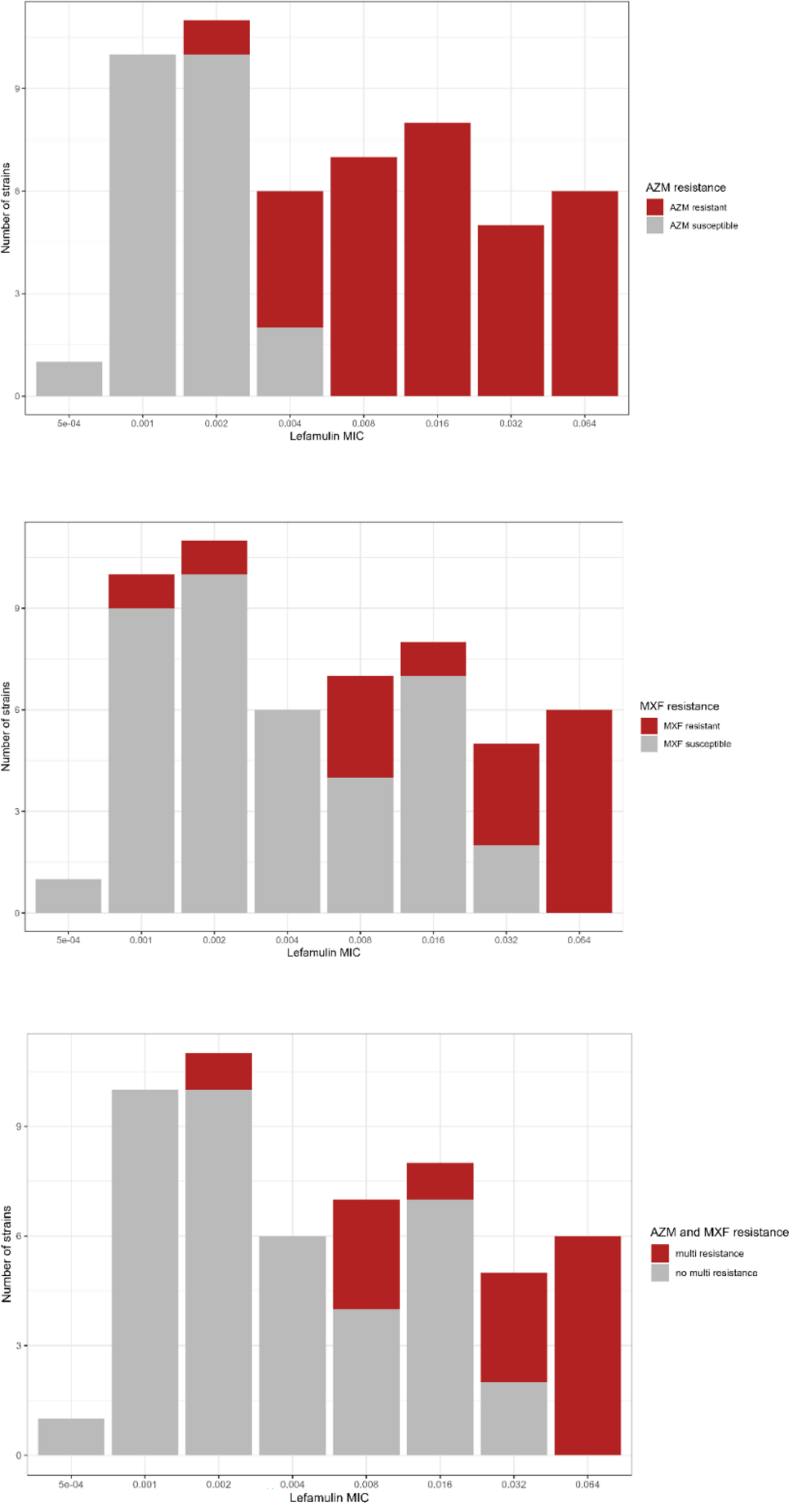
Distribution of lefamulin MICs for 54 *M*. *genitalium* isolates stratified by macrolide and quinolone resistance status. AZM, azithromycin; MXF, moxifloxacin.

Lefamulin was highly active *in vitro* against the 54 *M*. *genitalium* isolates tested (Fig. 1). The MICs ranged from 0.0005 to 0.064 µg/mL (Table 1). The median and modal MIC were 0.004 and 0.002 µg/mL, respectively, and the MIC_50_ and MIC_90_ were 0.004 and 0.064 µg/mL, respectively (Table 2). Lefamulin displayed bactericidal activity against all tested isolates, with MBC values ranging from 0.001 to 0.128 µg/mL (Table 1). The majority of MBC values (*n* = 40 of 54, 74.1%) were equal to the MICs or one- (*n* = 10, 18.5%) and twofold (*n* = 2, 3.7%) dilutions higher than the MIC (Table 1). MICs of the 54 *M*. *genitalium* isolates were also stratified by azithromycin and moxifloxacin susceptibility, and macrolide-susceptible isolates had significantly lower MICs than macrolide-resistant isolates (*P* < 0.001) (Table 2). The macrolide-resistant isolates were stratified by A2058G and A2059G mutations, and isolates carrying A2058G mutations had significantly lower MICs (*P* = 0.004) (Table 2). Also, the macrolide-resistant isolates were stratified by quinolone resistance, and isolates with dual resistance had significantly higher lefamulin MICs (*P* = 0.003) probably explained by the higher proportion of quinolone-resistant isolates with MRM in position 2059 (8 of 13, 62%). Quinolone-susceptible isolates displayed significantly lower lefamulin MICs than quinolone-resistant isolates (*P* < 0.001) (Table 2). However, since 14 of the 15 quinolone-resistant isolates were also macrolide resistant, no conclusion regarding a correlation of quinolone resistance with elevated lefamulin MICs can be made. The MICs for lefamulin were compared to the MICs for azithromycin, doxycycline, and moxifloxacin, respectively, and were significantly lower against both susceptible and resistant populations indicating that lefamulin was significantly more potent than the three antibiotics tested in this study (*P* < 0.001) (Table 2). Furthermore, the isolates were stratified based on low (<1 mg/L) and high (≥1 mg/L) doxycycline MIC (Table 2). Surprisingly, isolates with low doxycycline MICs tended to have significantly higher lefamulin MICs (Table 2). This was not due to interaction with MRMs, as the proportion of strains with A2059G mutation was similar between the groups, with 9 of 24 (37.5%) isolates in the high doxycycline group carrying an A2059G mutation associated with high lefamulin MIC, compared to 10 of 30 (33.3%) in the low doxycycline group (*P* = 0.781).

**TABLE 2:** MICs of 54 *Mycoplasma genitalium* isolates

Antibiotic	MIC_50_ (µg/mL)	MIC_90_ (µg/mL)	MIC range (µg/mL)	Modal MIC (µg/mL)	*P* value
Lefamulin (total, *n* = 54)	0.004	0.064	0.0005–0.064	0.002	
Lefamulin (azithromycin susceptible, *n* = 23)	0.002	0.002	0.0005–0.004	0.002	0.001^*a*^
Lefamulin (azithromycin resistant, *n* = 31)	0.016	0.064	0.002–0.064	0.016	
Lefamulin (A2058G, *n* = 9)	0.008	0.016	0.002–0.016	0.004	0.004^*a*^
Lefamulin (A2059G, *n* = 20)	0.32	0.064	0.004–0.064	0.064	
Lefamulin (moxifloxacin susceptible, *n* = 39)	0.002	0.016	0.0005–0.032	0.002	0.001^*a*^
Lefamulin (moxifloxacin resistant, *n* = 15)	0.032	0.064	0.001–0.064	0.064	
Lefamulin (dual resistance, *n* = 14)	0.032	0.064	0.002–0.064	0.064	0.003^*a*^
Lefamulin (doxycycline MIC < 1 mg/L, *n* = 30)	0.008	0.064	0.0005–0.064	0.002	0.014^*a*^
Lefamulin (doxycycline MIC ≥ 1 mg/L, *n* = 24)	0.004	0.016	0.001–0.032	0.001	
Azithromycin (total, *n* = 50)	16	64	0.001–128	64	0.001^*b*^
Azithromycin (azithromycin susceptible, *n* = 21)	0.001	0.004	0.001–0.016	0.001	
Azithromycin (azithromycin resistant, *n* = 29)	64	128	8–128	64	
Azithromycin (moxifloxacin susceptible, *n* = 35)	0.004	64	0.001–128	0.001	
Azithromycin (moxifloxacin resistant, *n* = 15)	64	64	0.002–128	64	
Doxycycline (total, *n* = 54)	0.5	2	0.125–2	0.5	0.001^*b*^
Doxycycline (azithromycin susceptible, *n* = 23)	0.5	2	0.125–2	0.5	
Doxycycline (azithromycin resistant, *n* = 31)	1	2	0.25–2	0.5	
Doxycycline (moxifloxacin susceptible, *n* = 39)	0.5	2	0.125–2	0.5	
Doxycycline (moxifloxacin resistant, *n* = 15)	1	2	0.5–2	1	
Moxifloxacin (total, *n* = 53)	0.125	4	0.03–16	1.125	0.001^*b*^
Moxifloxacin (azithromycin susceptible, *n* = 23)	0.125	0.25	0.03–16	0.125	
Moxifloxacin (azithromycin resistant, *n* = 30)	0.125	4	0.03–16	0.125	
Moxifloxacin (moxifloxacin susceptible, *n* = 39)	0.125	0.25	0.03–0.25	0.125	
Moxifloxacin (moxifloxacin resistant, *n* = 14)	4	16	0.5–16	4	

^*a*^ The Mann-Whitney test was applied for pairwise comparisons between groups.

^*b*^ The Wilcoxon signed-rank test was applied for comparisons across different antibiotics.

### Synergy of lefamulin and doxycycline

The synergy of lefamulin in combination with doxycycline was tested by checkerboard experiments using eight *M. genitalium* isolates based on the presence of ParC and/or 23S mutations. These included the *M. genitalium* G37 type strain (wild type), two isolates each with only macrolide resistance mediated by A2058G or A2059G mutations, and two isolates with ParC S83I and one with ParC D87N, all with macrolide resistance (Table 3). The FICI values of the eight *M. genitalium* isolates ranged from 0.5625 to 1.064 for lefamulin in combination with doxycycline (Table 3), indicating no synergy or antagonism according to the criteria of Odds (23). An additive effect was found for three strains that were resistant to macrolides and fluoroquinolones (M6270, M6489, and M6957) using the criteria of Doern (24).

**TABLE 3:** Determination of synergy between lefamulin and doxycycline against eight *M. genitalium* isolates expressed as the fractional inhibitory concentration index^*a*^^,^^*b*^

Strain	23S rRNA gene mutation	ParC mutation	GyrA mutation	FICI
G37	WT	WT	WT	1
M6270	A2059G	c186t silent	WT	0.625
M6320	A2059G	WT	WT	1
M6321	A2058G	WT	WT	1.064
M6489	A2059G	S83I	D99N	0.5625
M6735	A2059G	S83I	D99N	1
M6957	A2058G	P62S (NS)	WT	0.75
M6984	A2058G	D87N	ND	1

^*a*^ Synergy is defined as FICI ≤ 0.5 and antagonism as FICI > 4. Macrolide resistance mediating mutations in the 23S rRNA gene according to *Escherichia coli* numbering. ParC mutations leading to moxifloxacin resistance according to *M. genitalium* numbering.

^*b*^ WT, wild type; NS, non-significant mutation; ND, not done.

## DISCUSSION

The novel antimicrobial lefamulin was found to have potent activity against all 54 isolates of *M. genitalium* tested *in vitro* with all (100%) isolates inhibited at or below 0.064 µg/mL. The collection of isolates included a high proportion of quinolone- (28%) and macrolide-resistant (57%) isolates, with a high proportion of dual resistance. This makes them difficult to treat. The study is strengthened by this unique collection of *M. genitalium* isolates, originating from 49 individuals, with a wide range of resistance-mediating mutations and by the use of the Vero cell culture system, which allows cultivation of difficult-to-culture *M. genitalium* isolates. Although the Vero cell culture system is not validated and approved by international susceptibility testing organizations, results are generally in good agreement with the CLSI broth dilution assay, with the exception of macrolide MICs that tend to be slightly higher than with the conventional broth dilution assay (21). When comparing the lefamulin MIC distribution with that of azithromycin, doxycycline, and moxifloxacin, respectively, lefamulin was significantly more potent even against the susceptible subpopulations. Although the lefamulin MIC values were elevated for macrolide-resistant isolates, particularly for those with the A2059G mutation showing azithromycin MICs of up to 128 µg/mL, the lefamulin MIC values were ≤0.064 µg/mL, still very low and well below plasma and urogenital tissue concentrations that are typically achieved by the administration of the clinically available dosing of 600 mg oral and 150 mg IV, respectively, approved for the treatment of community-acquired pneumonia (10, 18). Moreover, lefamulin demonstrated bactericidal activity with MBC values being equal to the MIC or at maximum fourfold higher than the MIC value. These results are in good agreement with results from studies investigating the antibacterial activity of lefamulin against mycoplasmas *in vitro* and in the clinical setting, including *Mycoplasma pneumoniae* (11, 12, 25), *Mycoplasma hominis* (26), and *Mycoplasma amphoriphorme* (27).

Checkerboard experiments indicated no antagonism or synergy for lefamulin in combination with doxycycline (23) and an additive effect in three isolates using the criteria of Doern (24). The lack of antagonism is very important since tetracyclines are the only antimicrobials where *in vitro* resistance in *M. genitalium* is rare and has been demonstrated only once (28). However, when used as monotherapy, doxycycline is only effective in 30%–45% of cases (29); on the other hand, doxycycline has been shown to significantly decrease the *M. genitalium* load and decrease the selection of macrolide resistance during resistance-guided sequential therapy (30). Consequently, pre-treatment with doxycycline is recommended in several guidelines (31–33). A recent study investigating four different lefamulin treatment regimens in a small number of patients with *M. genitalium* found an overall modest efficacy of lefamulin; however, lefamulin was effective in some cases. The majority of the patients were heavily pre-treated, which might explain the low cure rates (34). The gap between *in vitro* efficacy and treatment efficacy in patients remains to be solved.

### Conclusion

Our findings suggest that lefamulin may represent a promising antimicrobial with potent bactericidal activity against *M. genitalium,* including isolates resistant to macrolides, fluoroquinolones, or both classes. Since new antimicrobials and antimicrobial combinations are urgently needed to treat an increasing number of patients with *M. genitalium* infection and prevent the further spread of multi-drug-resistant strains, studies evaluating the clinical efficacy of lefamulin in patients with *M. genitalium* infections are recommended.
